# Survivorship-Care-Programme für Krebspatienten: die Bedeutung von Risikostratifizierung, Selbstmanagement- und Gesundheitskompetenzen im Zeitalter digitaler Versorgung

**DOI:** 10.1007/s00103-022-03514-1

**Published:** 2022-03-11

**Authors:** Anja Mehnert-Theuerkauf, Peter Esser

**Affiliations:** Abteilung für Medizinische Psychologie und Medizinische Soziologie Universitätsklinikum Leipzig, Medizinische Fakultät, Universität Leipzig, Philipp-Rosenthal-Str. 55, 04103 Leipzig, Deutschland

**Keywords:** Psychoonkologie, Lebensqualität, Onkologische Versorgung, Überleben mit Krebs, Versorgungsforschung, Psycho-oncology, Quality of life, Cancer care, Cancer survivorship, Health services research

## Abstract

In Deutschland wie allen anderen Industrieländern weltweit nimmt mit einer älter werdenden Bevölkerung und einer verbesserten Krebsfrüherkennung, Diagnostik und onkologischen Behandlung die Zahl der Patienten, die geheilt werden oder lange Zeit mit der Erkrankung leben, deutlich zu (Cancer Survivors). Ein Leben mit und nach einer Krebserkrankung bedeutet für viele Patienten ein Leben mit körperlichen und psychosozialen krankheits- und behandlungsbedingten Langzeit- und Spätfolgen. Angesichts des demografischen Wandels, der steigenden Krebsprävalenz sowie des medizinischen Fortschritts ist eine der dringenden Fragen, wie eine qualitativ hochwertige individualisierte und gleichzeitig finanzierbare Krebsversorgung für älter werdende, häufig multimorbide Patienten sichergestellt werden kann.

Diese Entwicklungen erfordern neben einer Stärkung der Krebsprävention die Erforschung und Umsetzung einer individualisierten Nachsorge im Rahmen von Survivorship-Care-Programmen (SCP). Übergreifende Zielsetzung von SCP ist es, den körperlichen wie psychosozialen Langzeit- und Spätfolgen vorzubeugen bzw. diese zu minimieren, die Mortalität zu senken sowie die Lebensqualität von Patienten zu verbessern. Die Evidenz zur Wirksamkeit von SCP hinsichtlich der Verbesserung patientenberichteter Endpunkte ist bislang nicht eindeutig. Die Bereitstellung von maßgeschneiderten Informationen sowie von risikomodifizierenden und bedarfsorientierten Angeboten auf der Basis einer Risikostratifizierung wird als zentraler Bestandteil bei der Implementierung von SCP angesehen. Dabei nimmt die Förderung von Selbstmanagement- und Gesundheitskompetenzen der Patienten, insbesondere vor dem Hintergrund der Zunahme von digitalen Gesundheitsanwendungen, einen hohen Stellenwert ein.

## Abstract (en)

Due to an ageing population and improved early cancer detection, medical diagnostics and oncological treatment, the number of patients who are cured or live with the disease for a long time (cancer survivors) is increasing rapidly in Germany, as in all other industrialised countries worldwide. For many patients, living with and after cancer means living with physical and psychosocial disease and treatment-related long-term and late effects. In view of demographic change, rising cancer prevalence and medical progress, one of the urgent questions is how to ensure high-quality individualised and at the same time affordable cancer care for ageing patients with multimorbidity.

In addition to strengthening cancer prevention, these developments require research and implementation of individualised aftercare within the framework of survivorship care plans (SCPs). The overarching goal of SCPs is to prevent or minimise the physical and psychosocial long-term and late effects, reduce mortality and improve the patients’ quality of life. The evidence on the effectiveness of SCPs in improving patient-reported endpoints is so far not clear. The provision of tailored information as well as risk-modifying and demand-orientated offers based on risk stratification are seen as central components in the implementation of SCPs. In this context, the promotion of self-management and health literacy of patients also take on a high priority against the background of the increase in digital health applications.

## Hintergrund

In Deutschland wie allen anderen Industrieländern weltweit nimmt mit einer älter werdenden Bevölkerung und einer verbesserten Krebsfrüherkennung, Diagnostik und onkologischen Behandlung die Zahl der Patienten, die geheilt werden oder lange Zeit mit der Erkrankung leben, deutlich zu. Den Schätzungen des Global Organization Board of Cancer Association Network (GLOBOCAN) der International Agency for Research on Cancer (IARC) zufolge lag die weltweite krebsbezogene Inzidenz 2020 bei 19,3 Mio., die Mortalität bei etwa 10,0 Mio. Personen. Bis 2040 wird ein Anstieg der Inzidenz von 47 % gegenüber 2020 erwartet, wodurch weltweit mit etwa 28,4 Mio. Krebspatienten zu rechnen ist [1]. In Deutschland leben etwa 4,65 Mio. Menschen mit bzw. nach einer Krebserkrankung (Cancer Survivors), bei etwa 2 Dritteln von ihnen liegt die Krebsdiagnose bereits 5 oder mehr Jahre zurück [2].

In diesem Übersichtsartikel werden die besonderen Herausforderungen für eine individualisierte Nachsorge von Cancer Survivors im Rahmen von Survivorship-Care-Programmen (SCP) aufgezeigt, die Zielbereiche, Programminhalte und die internationale Studienlage zur Evidenz von SCP beschrieben sowie aktuelle Ansätze zur Risikostratifizierung vor dem Hintergrund der Förderung von Selbstmanagement- und Gesundheitskompetenzen dargestellt. Zugrunde gelegt wurden dabei aktuelle internationale Übersichtsarbeiten und systematische Reviews zu Qualitätskriterien von Cancer-Survivorship-Care-Programmen sowie zur Wirksamkeit und Umsetzung dieser Programme (u. a. [9, 17]).

### Cancer-Survivorship-Phasen

Der von Fitzhugh Mullan geprägte Begriff des „Cancer Survivorship“ [3] wurde in den letzten Jahren unterschiedlich abgegrenzt. International durchgesetzt hat sich dabei die Definition der US-amerikanischen Patientenorganisation National Coalition for Cancer Survivorship (NCCS), die jeden Patienten vom Zeitpunkt der Diagnose an bis zu seinem Tod als „Cancer Survivor“ – d. h. als Krebsüberlebenden – bezeichnet [4]. Diese Definition, der sich zahlreiche internationale Fachgesellschaften angeschlossen haben, soll vor allem Kliniker dazu ermutigen, die onkologische Versorgung entlang eines Kontinuums zu verstehen und die Auswirkungen der Krebstherapie auf die Lebensqualität der Patienten bereits frühzeitig bei der Diagnosestellung und Therapieplanung zu berücksichtigen. Spezifischere Definitionsvorschläge orientieren sich an Phasenmodellen, die die akute Behandlungsphase sowie das mittlere (bis 5 Jahre nach Diagnosestellung) und das Langzeitüberleben (in der Regel ab dem fünften Jahr nach Diagnosestellung) unterteilen [4]. Unterschieden werden darüber hinaus das krebsfreie Überleben und das Überleben mit einer chronischen oder fortschreitenden Krebserkrankung.

### Versorgungsbedarf der Langzeit- und Spätfolgen

Ein Leben mit und nach einer Krebserkrankung bedeutet in vielen Fällen ein Leben mit krankheits- und behandlungsbedingten Langzeit- und Spätfolgen, d. h. Auswirkungen der Erkrankung und Therapie, die lange Zeit anhalten oder erst spät in der Survivorship-Phase auftreten. Götze und Kollegen [5] zeigten an einer großen krebsregisterbasierten Studie mit 1002 Langzeitüberlebenden in Deutschland, dass die Patienten durchschnittlich über 5 komorbide Beschwerden berichteten; am häufigsten waren Fatigue, Schlaflosigkeit und Schmerzen. Sie wiesen weiterhin eine höhere körperliche Symptombelastung auf als eine alters- und geschlechtsadjustierte Stichprobe der Allgemeinbevölkerung. Langzeitüberlebende hatten weiterhin eine geringere Lebensqualität als die Allgemeinbevölkerung, insbesondere in den Dimensionen alltägliche Aktivitäten, soziales Leben, psychisches Wohlbefinden und finanzielle Schwierigkeiten.

In Abhängigkeit von der Krebsart und den tumorentitätsspezifischen Behandlungen unterscheidet sich die Symptombelastung zwischen den Populationen; typische körperliche Folgen sind dabei vor allem chronische Schmerzen, kardiovaskuläre, neurologische, endokrine Erkrankungen und Störungen sowie Zweitneoplasien ([6]; siehe auch den Beitrag von Ernst und Schilling in diesem Themenheft). Auf psychosozialer Ebene stehen psychische Belastungen wie Ängstlichkeit (u. a. Progredienzangst), Depressivität und eine eingeschränkte Lebensqualität im Vordergrund, aber auch Probleme bzgl. der Selbstständigkeit im Alltag, der Reintegration in das berufliche Leben, der sozialen Teilhabe, Partnerschaft und Familie sowie Aspekte eines gesunden Lebensstils und der Gesundheitsförderung ([7]; siehe auch den Beitrag von Weis in diesem Heft).

Bereits heute entfallen ca. 7 % der insgesamt mit 338 Mrd. € bezifferten Krankheitskosten in Deutschland auf Krebserkrankungen [8]. Angesichts des demografischen Wandels in unserer Bevölkerung, der steigenden Krebsprävalenz sowie der Fortschritte in der Biomedizin ist eine der dringenden Fragen, wie eine qualitativ hochwertige, zunehmend kostenintensive, individualisierte Krebsversorgung für älter werdende, häufig multimorbide Patienten sichergestellt werden kann. Diese Entwicklungen erfordern zum einen eine wirksamere Implementierung von Krebspräventionsmaßnahmen einschließlich Sekundär- und Tertiärprävention, zum anderen die Erforschung und Umsetzung einer qualitativ hochwertigen Versorgung der Patienten in allen Survivorship-Phasen mit dem Ziel, das Überleben und das Management der körperlichen und psychischen Langzeit- und Spätfolgen zu verbessern sowie die psychosozialen Auswirkungen zu lindern [9].

### Stärkung der Prävention, Gesundheitskompetenz und Lebensqualität in der Nationalen Dekade gegen Krebs und im Europäischen Krebsplan

Nach aktuellen Schätzungen des Deutschen Krebsforschungszentrums (DKFZ) kann das Risiko für eine Krebsneuerkrankung durch eine gesündere Lebensweise, d. h. überwiegend durch Verhaltensfaktoren, deutlich reduziert werden [10–12]. Zu den wichtigsten vermeidbaren Risikofaktoren zählen Tabakkonsum und Alkoholabusus, Übergewicht, falsche Ernährung und Bewegungsmangel, UV-Strahlung insbesondere aus Solarien, Infektionen (z. B. mit humanen Papillomviren – HPV) sowie spezifische Umweltfaktoren (u. a. Feinstaub). Diese Faktoren sind dabei nicht nur im Sinne der Primärprävention und Sekundärprävention von Krebserkrankungen bzw. bei Risikopopulationen (z. B. Frauen mit *BRCA*-Genmutationen, die ein Risiko für Mamma- und Ovarialkarzinom darstellen) hoch relevant, sondern auch im Rahmen der Tertiärprävention durch die Verhinderung oder Abmilderung von Krankheitsfolgen in der Survivorship-Phase.

Sowohl die Nationale Dekade gegen Krebs (NDK), die 2019 vom Bundesministerium für Bildung und Forschung (BMBF) gemeinsam mit dem Bundesministerium für Gesundheit (BMG; [13]) ausgerufen wurde, als auch der 2021 veröffentlichte Europäische Krebsplan (Europe’s Beating Cancer Plan; [14]) verfolgen klare Zielsetzungen hinsichtlich der Verbesserung der Primär‑, Sekundär- und Tertiärprävention von Krebserkrankungen und deren Folgen sowie der Verbesserung der Qualität der onkologischen Versorgung und der Lebensqualität der Betroffenen in allen Survivorship-Phasen. Diese Zielsetzungen gehen einher mit einem stärkeren Einbezug der Patienten selbst, d. h. der Förderung von Gesundheit durch den eigenen Lebensstil und der Steigerung von Gesundheitskompetenz (Health Literacy). Zu diesem Einbezug gehört nicht zuletzt auch die Beteiligung von Patienten in Forschungsprojekten und bei der Implementierung von wissenschaftlichen Ergebnissen in die Versorgung.

## Survivorship-Care-Programme (SCP)

### Übergreifende Zielsetzungen

In den letzten Jahren gab es national wie international zahlreiche Initiativen, die vielfältigen Anforderungen an die onkologische Versorgung und Nachsorge in Empfehlungen für eine bessere Versorgung Krebskranker u. a. im Rahmen von Survivorship-Care-Programmen umzusetzen [13–16]. Übergreifende Zielsetzung ist es, die Mortalität zu senken, den körperlichen wie psychosozialen Langzeit- und Spätfolgen vorzubeugen bzw. diese zu minimieren sowie die Lebensqualität von Patienten zu verbessern [15].

Die American Society of Clinical Oncology (ASCO) hat bereits 2013 Kriterien für eine qualitativ hochwertige Cancer-Survivorship-Versorgung publiziert [16]. Diese umfassen u. a.:

- die Bereitstellung von Screeningempfehlungen in Bezug auf Rezidive/Zweittumoren,
- das Monitoring und Management von psychosozialen und medizinischen Spätfolgen,
- die Bereitstellung von umfassenden Informationen für Patienten hinsichtlich der Diagnose, Behandlungsexpositionen und möglicher Spät- und Langzeitfolgen,
- die Abschätzung des familiären genetischen Risikos (falls erforderlich),
- die Beratung über Ernährung, Bewegung und gesundheitsfördernde Maßnahmen,
- die Bereitstellung von Ressourcen zur Unterstützung bei finanziellen und versicherungstechnischen Fragen sowie
- die Stärkung und Befähigung (Empowerment) der Patienten, sich für ihre eigenen Gesundheitsbedürfnisse stärker einzusetzen.

### Ausgestaltung von SCP

Survivorship-Care-Programme sind mit Blick auf die internationale Versorgungslandschaft formal wie inhaltlich sehr unterschiedlich ausgestaltet. Die meisten Forschungsarbeiten zu SCP kommen aus dem Vereinigten Königreich, Kanada, den USA, den Niederlanden und Schweden sowie Australien [17]. SCP unterscheiden sich u. a. im Spezialisierungsgrad der Behandler und in der Art der multidisziplinären Ausgestaltung bzw. Implementierung, in der Art der Koordination und Kooperation sowie der primär für das Programm verantwortlichen Personen [16, 18]. Grundsätzlich lassen sich fachärztlich geleitete SCP (u. a. durch Onkologen/Hämatoonkologen und/oder Hausärzte) von interdisziplinär koordinierten SCP (z. B. im Rahmen einer integrativen Survivorship-Klinik) unterscheiden, die unterschiedliche Fachdisziplinen vereinen und bspw. von einer spezialisierten Fachpflege geleitet werden [16, 18]. Das fachärztlich geführte SCP im Sinne der klassischen Krebsnachsorge ist international das am weitesten verbreitete Modell und zielt auf eine vor allem medizinische Versorgung mit dem Fokus auf die frühzeitige Erkennung von Rezidiven oder Zweittumoren.

### Identifikation relevanter Zielbereiche und Programminhalte von SCP

Nekhlyudov und Kollegen [9] entwickelten auf der Grundlage umfassender Interviews mit verschiedenen Experten (u. a. Wissenschaftlern und Klinikern aus den Bereichen der Primärversorgung, Onkologie und Psychologie), politischen Entscheidungsträgern, Kostenträgern, Interessenverbänden und Patientenvertretern ein Rahmenkonzept für eine qualitativ hochwertige Versorgung von Krebspatienten in der Survivorship-Phase. Als zentrale Komponenten wurden 5 Bereiche identifiziert, die sich in überwiegend krebs- und behandlungsspezifische (1–3) sowie allgemeine gesundheitsrelevante Aspekte (4–5) unterteilen lassen (siehe Infobox 1).

Als Ergebniskriterien zur Bewertung der Wirksamkeit von Cancer-Survivorship-Care-Programmen benennen Nekhlyudov und Kollegen [9] neben der Lebensqualität als multidimensionales Konstrukt und der Funktionsfähigkeit auch Aspekte wie die Inanspruchnahme des Gesundheitswesens, insbesondere Notfallversorgung, intensivmedizinische Versorgung und Krankenhausaufenthalte, sowie die Mortalität und die Kosten der Versorgung.

### Zur Effektivität von SCP

Bisherige Studienergebnisse über die Wirksamkeit von SCP in verschiedenen Cancer-Survivorship-Populationen sind heterogen und zeigen vereinzelt positive Effekte in Hinblick auf verschiedene Ergebniskriterien. Insgesamt ist die Evidenz hinsichtlich der Verbesserung patientenberichteter Endpunkte jedoch nicht eindeutig [27–29]. Dennoch zeigen Studien auch, dass SCP von Patienten als auch von Experten überwiegend positiv eingeschätzt und von zahlreichen Patientenorganisationen und Fachgesellschaften empfohlen werden [28, 29].

In Anlehnung an die dargelegten Zielbereiche von SCP nach Nekhlyudov und Kollegen [9] liegen erste Befunde aus systematischen Übersichtsarbeiten zur vergleichenden Effektivität verschiedener Formen von SCP vor [17]. Dabei gingen verschiedene Konzepte von eher fachärztlich koordinierten oder interdisziplinär bzw. pflegerisch geleiteten SCP in die Analysen ein.

In Hinblick auf die Zielsetzung *Prävention und Überwachung der primären Krebserkrankung und potenziell neu auftretender Krebsarten* zeigen die Studien keine signifikanten Unterschiede zwischen verschiedenen SCP-Modellen in Bezug auf die Inzidenz von Rezidiven oder die Zeit bis zur Erkennung eines Rezidivs. Systematische Übersichtsarbeiten zur Effektivität von SCP hinsichtlich der Identifikation neu auftretender Krebsarten liegen nicht vor [17].

Auch hinsichtlich der *Überwachung, Behandlung und Versorgung körperlicher Erkrankungs- und Behandlungsfolgen* ist die Effektivität unterschiedlicher SCP-Modelle unklar [17], auch wenn sich vereinzelt Vorteile von hausärztlich sowie pflegerisch koordinierten SCP-Modellen in der Reduktion von Fatigue zeigten [30, 31] sowie vereinzelt Vorteile von fachärztlich geleiteten SCP-Modellen in der Reduktion von Appetitverlust [30]. Keine signifikanten Unterschiede in der Effektivität verschiedener SCP-Modelle wurden bezüglich der *Überwachung, Behandlung und Versorgung psychosozialer Erkrankungs- und Behandlungsfolgen* wie Angst, Depressivität oder Distress gefunden [17].

In Bezug auf die Zieldimension *Überwachung, Behandlung und Versorgung chronischer Erkrankungen und Gesundheitseinschränkungen* liegen keine systematischen Übersichtsarbeiten vor, die einen Wirksamkeitsvergleich verschiedener SCP-Modelle gestatten [17]. Auch Studien bzw. systematische Übersichtsarbeiten zur differenziellen Effektivität von verschiedenen SCP mit Blick auf die Zieldimension *Gesundheitsförderung und (Sekundär‑/Tertiär‑)Prävention* liegen nur vereinzelt vor [17, 31]. Diese deuten auf einen Vorteil interdisziplinärer SCP hinsichtlich der Verbesserung des Lebensstils hin [32].

Weitere relevante Zielgrößen wie Indikatoren einer patientenzentrierten Kommunikation und gemeinsamen Entscheidungsfindung oder Selbstmanagementfähigkeiten wurden bislang nicht systematisch untersucht, ebenso wie der Effekt von Versorgungsstrukturen innerhalb des SCP (u. a. Nutzung von Gesundheitsinformationstechnologien), weshalb keine vergleichenden Analysen durchgeführt werden konnten [17]. Allerdings weisen vereinzelte Studien auf einen Vorteil von hausärztlichen sowie interdisziplinär bzw. pflegerisch koordinierten SCP hinsichtlich einer besseren interdisziplinären Kommunikation und Koordination der Versorgung hin [33]. Pflegerisch koordinierte SCP scheinen darüber hinaus signifikant positiv mit einer höheren Lebensqualität der Patienten in Zusammenhang zu stehen [34]. Unterschiede zwischen hausärztlichen sowie interdisziplinär bzw. pflegerisch koordinierten SCP hinsichtlich der Mortalität gemessen anhand der Inzidenz von Todesfällen oder Überlebensraten wurden ebenfalls nicht gefunden [17].

## Risikostratifizierung, Gesundheitskompetenz und Selbstmanagement

Die Bereitstellung von maßgeschneiderten Informationen sowie von risikomodifizierenden und bedarfsorientierten Angeboten ist ein zentraler Bestandteil im Rahmen der Implementierung von SCP. Angesichts der Vielzahl körperlicher und psychosozialer Risiken und Beeinträchtigungen stellen die Erfassung und Beurteilung individueller Risikokonstellationen eine Herausforderung dar. Auch vor dem Hintergrund der Kosteneffizienz angesichts der steigenden Prävalenz von Krebserkrankungen bei einer älter werdenden Bevölkerung nehmen die Förderung der Gesundheitskompetenz, des Selbstmanagements und digitale Versorgungskonzepte eine zunehmend wichtige Rolle ein.

SCP sind bislang überwiegend informationsbasiert und enthalten in der Regel Informationen zur Krebsart, zu onkologischen Therapien und deren potenziellen Langzeit- und Spätfolgen, Informationen über die medizinische Nachsorgeplanung, die Rehabilitation, die psychosozialen Unterstützungsmöglichkeiten (u. a. Krebsberatung) sowie zu Empfehlungen bezüglich einer gesunden Lebensführung. Wie die Evidenzlage zur Effektivität von SCP zeigt, sind Informationen zwar eine wichtige Voraussetzung für eine erfolgreiche Implementierung, sie reichen aber bei Weitem nicht aus, um patientenrelevante Endpunkte zu verbessern, d. h. die Lebensqualität zu erhalten bzw. zu steigern und die Morbidität und Mortalität zu verringern. Notwendig scheinen eine Risikostratifizierung auf der Grundlage epidemiologischer Daten und eine bedarfsgerechte Planung der SCP-Inhalte im Sinne einer individualisierten Survivorship-Care-Planung, weiterhin die gezielte, aktive Förderung von Gesundheits- und Selbstmanagementkompetenzen [35].

### Risikostratifizierung

Für die Implementierung von SCP sind die Bewertung sowohl des individuellen Risikos für potenzielle negative medizinische und psychosoziale Folgen als auch die Berücksichtigung patientenseitiger Unterstützungsbedürfnisse erforderlich. Neben der eigentlichen Risikobewertung sind auch die Einschätzung verfügbarer wirksamer Interventionen sowie die gesundheitsökonomischen Auswirkungen relevant [35]. Watson und Kollegen [35] schlugen folgende Aspekte vor, die in eine Risikobewertung einfließen könnten:

1. *körperliche Gesundheit*: Wiederauftreten der Krebserkrankung, Zweittumoren, körperliche Langzeit- und Spätfolgen, Auswirkungen auf Komorbiditäten und Mortalität,
2. *psychische Gesundheit*: Depressivität, erhöhte Angst (einschließlich Progredienzangst), psychosexuelle Probleme, Lebensqualität,
3. *soziale Aspekte*: finanzielle Probleme, Arbeit/Beruf, Aus‑/Fort‑/Weiterbildung, zwischenmenschliche Probleme und soziale Interaktionen, Behinderung.

Eine Herausforderung stellt allerdings die Risikostratifizierung selbst dar, da für die Mehrzahl der genannten Bereiche epidemiologische Daten fehlen und die Langzeit- und Spätfolgen darüber hinaus umfassend und komplex sind. Darüber hinaus stellt die valide Erfassung spezifischer Risikokonstellationen in einem alltagstauglichen Screening eine weitere Hürde dar, zumal sowohl Risiken als auch patientenseitige Bedürfnisse im Verlauf der Krebserkrankung bzw. in der Survivorship-Phase variieren dürften. Denkbar wäre z. B. ein Kerninstrument, das zum einen die für die meisten Krebsarten relevanten Risiken abdeckt, zum anderen typische psychosoziale Belastungen erfasst. Ein solches Kerninstrument könnte durch krebsartspezifische oder patientengruppenspezifische (z. B. geriatrische) Informationen nach Bedarf erweitert werden, die dann in die Nachsorgestrategie in Kooperation mit dem Patienten eingehen.

Watson und Kollegen [35] schlagen die Zeit nach der Diagnose vor Beginn der Primärbehandlung als ersten Zeitpunkt für eine Risikostratifizierung auf der Grundlage des (zu erwartenden) Nebenwirkungsprofils und psychosozialer Aspekte vor. Weitere Zeitpunkte wären der Abschluss der Primärbehandlung, 6 und 12 Monate nach Primärbehandlung sowie jährliche Folgescreenings. Die Risikostratifizierung wird durch eine Bedarfserfassung ergänzt bzw. zwischenzeitlich ersetzt.

Entsprechend dem individuellen Risiko und den patientenseitigen Unterstützungsbedürfnissen stehen bei niedrigem (körperlichen und psychosozialem) Risiko und geringen Unterstützungsbedürfnissen Information und Selbstmanagement im Vordergrund. Patienten mit einem mittleren Risiko erhalten neben Informationen eine engmaschigere SCP-Versorgung und Patienten mit einem hohen Risiko eine komplexe Versorgung durch spezialisierte Dienste [35]. Wie dies für spezifische SCP konkret ausgestaltet werden kann, sollte im Rahmen von Ansätzen der Versorgungsforschung untersucht werden. Eine Koordination der Versorgung zwischen akutstationären, rehabilitativen und ambulanten (u. a. onkologischen, hausärztlichen, psychosozialen) Angeboten ist dabei von hoher Relevanz.

### Selbstmanagement- und (digitale) Gesundheitskompetenz

Ein zentrales Ziel von SCP ist die Verbesserung des gesundheitsbezogenen Selbstmanagements. Dabei sollen Patienten in die Lage versetzt werden, möglichst selbstständig mit ihrer Erkrankung und den Krankheitsfolgen umzugehen. Dazu gehören Ressourcenaktivierung und Selbststeuerung, der Erwerb effektiver Problemlösestrategien sowie die Inanspruchnahme von Unterstützungsangeboten [36, 37]. So nehmen im Rahmen von SCP Selbstmanagementansätze, die diese „Hilfe zur Selbsthilfe“ fördern und als zentrale Zielkriterien definieren, einen wichtigen Stellenwert ein [18]. Studien zeigen positive Effekte der Förderung von Selbstmanagementstrategien im Rahmen von SCP auf die psychische Belastung [38], die Qualität der Studien ist insgesamt aber begrenzt und der Forschungsbedarf hoch [39].

Eine wichtige Voraussetzung des Selbstmanagements ist die Gesundheitskompetenz, d. h. das Wissen, die Motivation und das Ausmaß, in dem ein Mensch in der Lage ist, relevante Gesundheitsinformationen und -dienste zu finden, zu verstehen, zu beurteilen und anzuwenden, um angemessene Gesundheitsentscheidungen zu treffen, die die Lebensqualität erhalten oder verbessern [40]. Im Nationalen Aktionsplan Gesundheitskompetenz [41] wurde die besondere Relevanz der Förderung von Gesundheitskompetenz bei Patienten mit chronischen Erkrankungen betont. Dass Gesundheitskompetenz positiv mit gesundheitsförderlichem Verhalten sowie mit selbstberichteter psychischer und körperlicher Gesundheit in Zusammenhang steht, wurde bereits bei nichtonkologischen Patientengruppen vielfach gezeigt [42, 43]. Gesundheitskompetenz scheint sich aber auch positiv auf die Wirksamkeit von E‑Health-Angeboten zur Verbesserung der Selbstmanagementkompetenzen bei Krebspatienten auszuwirken [44].

Im Zuge der technologischen Entwicklungen schließt Selbstmanagement immer mehr auch digitale Angebote mit ein. Digitale Gesundheitstechnologien wie die Anwendung von tragbaren Sensoren, Applikationen (Apps), sozialen Medien und Technologien zur Standortbestimmung ermöglichen die Erfassung und Nutzung von Daten, die für die Diagnosestellung, Prävention und Behandlung von Krankheiten wie für das individuelle Wohlbefinden von Patienten hohe Relevanz haben [45]. Digitale Gesundheitsangebote, darunter Videokonsultationen, elektronische Visiten oder digitale Patientenportale sind mit zunehmender Geschwindigkeit verfügbar und haben an Popularität gewonnen, da sie verschiedene Vorteile für Patienten bieten. Dazu gehören (a) ein besserer und zeitnaher Zugang zu evidenzbasierten gesundheitsrelevanten Informationen, (b) eine erhöhte Adhärenz/Compliance zum Beispiel durch Erinnerungen an die Einnahme von Medikamenten oder Arztterminen, (c) die schnellere und zeitnahe Messung von körperlichen und psychischen Symptomen und des Befindens, (d) ein besseres Verständnis über individuelle Reaktionen und Verläufe von Symptomen und des Befindens, (e) eine frühzeitige Initiierung von individuellen Strategien zur Aufrechterhaltung des Gesundheitsverhaltens sowie (f) die Versorgung in einem frühen bzw. früheren Stadium im Krankheitsverlauf [45]. Verfügbare Daten und grafische Darstellungen des subjektiven Zustands können die Patientenerfahrung validieren und dazu beitragen, die Zusammenarbeit mit Gesundheitsdienstleistern in der Survivorship-Phase zu verbessern. Darüber hinaus haben mobile Gesundheitsanwendungen das Potenzial, soziale und gesundheitliche Ungleichheiten zum Beispiel zwischen der urbanen und der ländlichen Bevölkerung zu verringern, wobei Letztere zuweilen unterversorgt ist [45].

Auch wenn digitale Gesundheitsangebote verschiedene Vorteile bieten, sind die förderlichen Faktoren sowie die Barrieren der Nutzung dieser Angebote noch nicht vollständig verstanden. Ernsting und Kollegen [46] untersuchten in einer bevölkerungsbasierten Studie die Zusammenhänge zwischen demografischen und gesundheitsbezogenen Faktoren, häufigen chronischen Erkrankungen und dem Gesundheitsverhalten bei der Nutzung digitaler Gesundheitsangebote. Die Ergebnisse deuten auf signifikante altersbedingte, sozioökonomische und gesundheitliche Einflüsse im Nutzungsverhalten hin. Die Vorteile der Anwendung digitaler Gesundheitstechnologien scheinen dabei insbesondere mit der digitalen Gesundheitskompetenz in Zusammenhang zu stehen [46], ein Ergebnis, das in anderen Studien bestätigt wurde [45]. Digitale Gesundheitskompetenz (E-Health Literacy) wird dabei definiert als die „Fähigkeit zum Suchen, Finden, Verstehen und Bewerten von Gesundheitsinformationen auf der Grundlage digitaler Quellen und das gewonnene Wissen so anzuwenden, um gesundheitliche Herausforderung zu adressieren und Probleme zu lösen“ [47, 48].

## Fazit

In den letzten Jahren wurden deutliche Fortschritte in Hinblick auf die Implementierung und Erprobung verschiedener Modelle von Survivorship-Care-Programmen (SCP) erzielt. Dennoch zeigt die Evidenzlage bezüglich ihrer Wirksamkeit in Hinblick auf die Verbesserung der Gesundheit und der Lebensqualität von Cancer Survivors, dass weiterhin erhebliche Anstrengungen vor allem in der Versorgungsforschung erforderlich sind, um SCP zu optimieren. Die Forschung zeigt insbesondere ein Defizit bei individualisierten SCP, die auf spezifische Gruppen von Cancer Survivors zugeschnitten sind und auf einem risikobasierten Ansatz mit wirksamen Elementen basieren. Darüber hinaus ist für eine erfolgreiche Implementierung von SCP im digitalen Zeitalter die Förderung der (digitalen) Gesundheitskompetenz unerlässlich. Spezialisierte Trainingsprogramme können dabei die Gesundheitskompetenz verbessern, z. B. bei älteren Patienten oder solchen mit niedrigem Bildungsniveau [49]. In diesen Programmen werden verschiedene Techniken der Informationsvermittlung angewandt, z. B. das gemeinsame Lernen, wobei die Teilnehmenden in Gruppen zusammenarbeiten, um sich im Rahmen der gemeinsamen Lösungsfindung ein umfassendes Verständnis anzueignen. Insgesamt besteht ein Mangel an hochqualitativen Studien und theoriebasierten Schulungsprogrammen, insbesondere bei Krebspatienten [49]. Bisherige Programme zeigen aber positive Effekte z. B. in Bezug auf das internetbezogene Wissen bzw. auf internetbezogene Fertigkeiten [49]. Gezielte Schulungsprogramme zur Verbesserung digitaler Gesundheitskompetenz können darüber hinaus helfen, das Risiko einer „digitalen Kluft“ (Digital Divide) zu reduzieren, d. h. einer Spaltung von Bevölkerungsgruppen durch unterschiedliche Zugangsmöglichkeiten zu Informations- und Kommunikationstechnologien, die zu einer Ungleichheit in der medizinischen Versorgung führen könnte. Weitere Forschung zur Wirksamkeit von SCP ist also notwendig, um eine breitflächige Implementierung zu ermöglichen.

### Infobox 1: Zentrale Komponenten von Survivor-Care-Programmen für Krebspatienten, nach Nekhlyudov et al. [9]

**1. (Sekundär‑/Tertiär‑)Prävention und Überwachung der primären Krebserkrankung und neu auftretender Krebsarten**: Krebspatienten haben ein Risiko für Rezidive und die Entwicklung von Zweittumoren, die auf eine genetische Veranlagung, Lebensgewohnheiten und/oder die Behandlungsexposition zurückzuführen sind [4, 19]. *Survivorship-Programme* sollten solche Risikofaktoren krankheitsspezifisch definieren, multidimensional bewerten (Familienanamnese, genetische Beratung und Tests, psychosoziale Anamnese, Behandlungsexposition) und darauf aufbauend evidenzbasierte und bedarfsabhängig risikomodifizierende Strategien des Monitorings implementieren (u. a. körperliche Untersuchung, Laboruntersuchung und/oder Bildgebung).

**2. Überwachung, Behandlung und Versorgung körperlicher Erkrankungs- und Behandlungsfolgen**: Krebspatienten haben ein erhöhtes Risiko für eine Vielzahl unterschiedlicher und häufig stark beeinträchtigender körperlicher Folgeprobleme [4, 19]. *Survivorship-Programme* sollten Art und Schweregrad der körperlichen Folgeprobleme systematisch erfassen und bewerten (u. a. Anamnese, körperliche Untersuchung und/oder validierte Instrumente) und darauf aufbauend evidenzbasierte Versorgungs‑, Behandlungs- und ggf. risikomodifizierende Strategien implementieren.

**3. Überwachung, Behandlung und Versorgung psychosozialer Erkrankungs- und Behandlungsfolgen**: Krebspatienten haben ein Risiko für eine Vielzahl unterschiedlicher und häufig stark beeinträchtigender psychosozialer Folgeprobleme [7, 20, 21].* Survivorship-Programme* sollten Art, Ausmaß und Schweregrad der psychosozialen Folgeprobleme systematisch erfassen und bewerten (u. a. Sozialanamnese, Psychodiagnostik und/oder validierte Instrumente) und darauf aufbauend evidenzbasierte Versorgungs- und Behandlungsansätze (u. a. psychosoziale Beratung, Psychoedukation, psychotherapeutische Interventionen) implementieren.

**4. Überwachung, Behandlung und Versorgung chronischer Erkrankungen und Gesundheitseinschränkungen**: Bei Krebspatienten besteht eine hohe Prävalenz von (z. T. altersbedingten) körperlichen und psychosozialen Erkrankungen und Gesundheitsbeeinträchtigungen, die bereits vor der Krebserkrankung bestanden haben oder nach Krebsbehandlung neu auftreten und sowohl die Multimorbidität als auch die Mortalität erhöhen können [22, 23]. *Survivorship-Programme* sollten bereits bestehende oder während bzw. nach der Krebserkrankung aufgetretene diagnostizierte körperliche und psychische chronische Erkrankungen (u. a. Bluthochdruck, Diabetes mellitus, Depression) miterfassen und im Rahmen der Behandlungs- und Nachsorgeplanung berücksichtigen.

**5. Gesundheitsförderung und (Sekundär‑/Tertiär‑)Prävention:** Viele Krebspatienten weisen auch in der Survivorship-Phase eine Reihe von risikoreichen Verhaltensweisen auf wie Tabak- und/oder übermäßigen Alkoholkonsum, Übergewicht und Bewegungsmangel [24, 25], die wiederum mit einer erhöhten Morbidität und Mortalität in Zusammenhang stehen [26]. *Survivorship-Programme* sollten im Sinne der Sekundär- und Tertiärprävention Lebensstilfaktoren und ungesunde Lebensgewohnheiten erfassen und spezifische, evidenzbasierte Beratungs- und Interventionsangebote zur Gesundheitsförderung (u. a. Tabakentwöhnung, Ernährungsberatung, Sport- und Bewegungsprogramme) zur Verfügung stellen. Dazu gehören u. a. auch Vorsorgeuntersuchungen für weitere chronische Erkrankungen oder Impfungen.
